# Assessing Asiatic cheetah’s individual diet using metabarcoding and its implication for conservation

**DOI:** 10.1038/s41598-022-15065-1

**Published:** 2022-07-06

**Authors:** Leili Khalatbari, Bastian Egeter, Hamed Abolghasemi, Ehsan Hakimi, Taher Ghadirian, Amir Hosein Khaleghi Hamidi, Houman Jowkar, Urs Breitenmoser, José Carlos Brito

**Affiliations:** 1 CIBIO, Centro de Investigação Em Biodiversidade e Recursos Genéticos, InBIO Laboratório Associado, Campus de Vairão, Universidade Do Porto, 4485-661 Vairão, Portugal; 2 Departamento de Biologia, Faculdade de Ciências, Universidade Do Porto, 4099-002 Porto, Portugal; 3 BIOPOLIS Program in Genomics, Biodiversity and Land Planning, CIBIO, Campus de Vairão, 4485-661 Vairão, Portugal; 4 Mohitban Society, No. 91, Moghaddas Ardebili str., Tehran, 19859-14747 Iran; 5 NatureMetrics, 1 Occam Court, Surrey Research Park, Guildford, GU2 7HJ UK; 6 Freelance Conservationist, No.1, Alley 25, Heydarpour st., Motahari blvd., Rafsanjan, Kerman, 7718936846 Iran; 7 I.R. Iran Department of Environment, Conservation of Asiatic Cheetah Project, Pardisan Park, Hemmat Highway, Tehran, 11396 Iran; 8 IUCN/SSC Cat Specialist Group, c/o KORA, 3074 Muri, Switzerland

**Keywords:** Conservation biology, Ecological genetics

## Abstract

Knowledge on diet composition allows defining well-targeted conservation measures of large carnivores. Little is known about ecology of critically endangered Asiatic cheetah, especially the overall diet and its possible regional differences. We used cheetah scats, metabarcoding technique and microsatellite markers to assess the individual and overall diet composition of the species across its entire range in Asia. Cheetahs were primarily predating on mouflon; following by ibex, cape hare and goitered gazelle. Despite their high availability, small-sized livestock was never detected. Goitered gazelles were only detected in an area where the habitat is mainly flatlands. In hilly areas, mouflon was the most frequent prey item taken. Ibex was typically taken in rugged terrain, but mouflon was still the most frequently consumed item in these habitats. High consumption of mouflon in comparison to goitered gazelle suggests that human pressure on lowland habitats has possibly forced Asiatic cheetahs to occupy suboptimal habitats where gazelles are less abundant. The protection of flatlands and the removal of livestock from them are needed to ensure the long-term survival of Asiatic cheetah. The laboratory and bioinformatics pipelines used in this study are replicable and can be used to address similar questions in other threatened carnivores.

## Introduction

Intensive human activities have resulted in the decline of global biodiversity including mammals^1,2^. Large carnivores are one of the groups that are globally threatened by population decline, range contraction and habitat fragmentation^3^. Conservation measures grounded in scientific studies can reduce these negative effects and allow carnivore populations to recover. Availability of prey is one of the primary factors related to carnivore abundance^4^. Thus, precise knowledge on diet composition and prey availability are needed to define appropriate conservation decisions.

**Figure 1:**
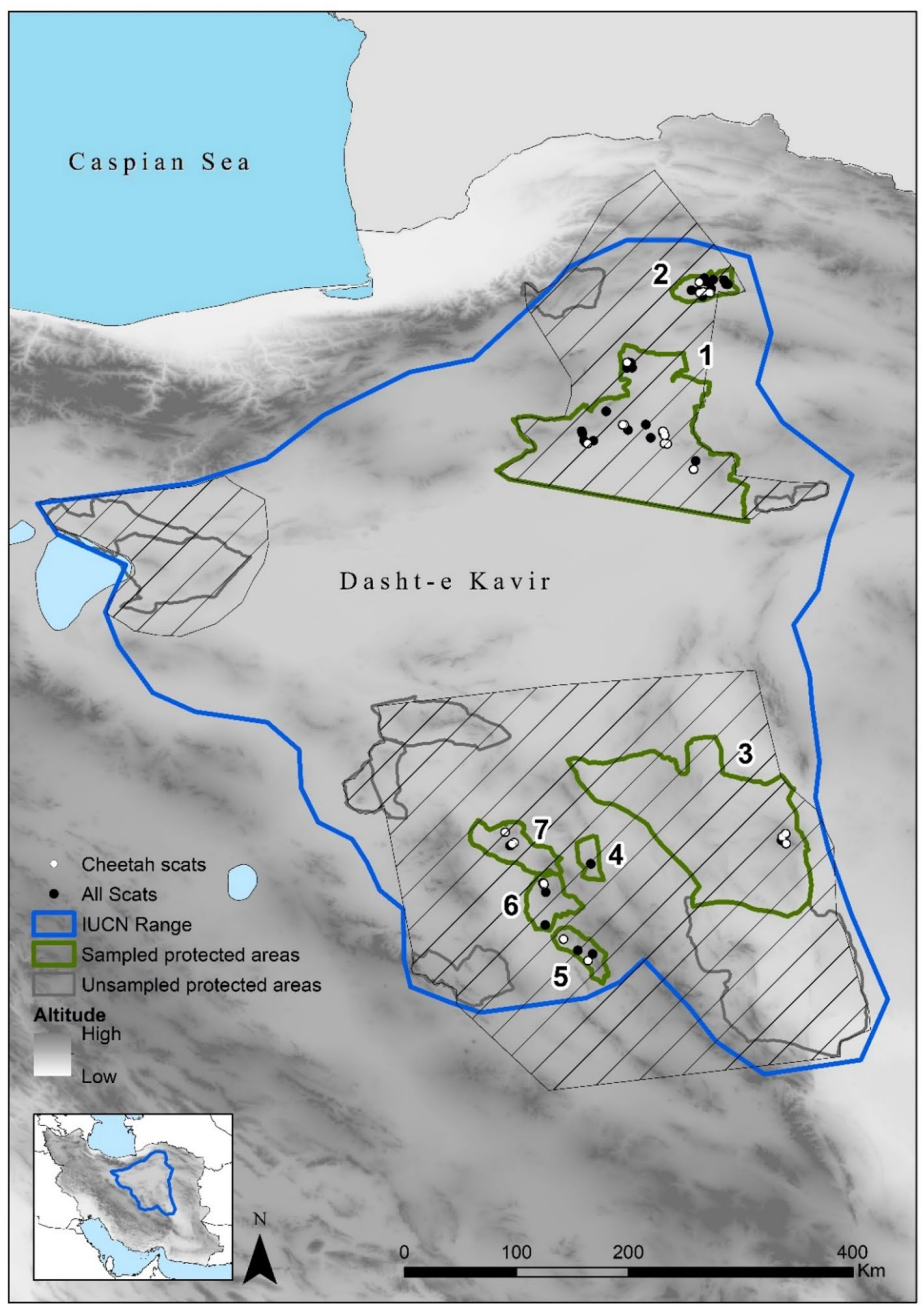
Current distribution of Cheetah in Iran (small inset) and location of subpopulations/protected areas from which samples were available for this study. The dashed lines depict the estimated boundaries of each subpopulation: Northern in the north, Southern in the south and Kavir in the west. Northern Subpopulation: (1) Touran Biosphere Reserve, (2) Miandasht WR and Zamen-e Ahoo NP; Southern subpopulation: (3) Naybandan WR; (4) Kamki Bahabad HPA, (5) Bafgh PA, (6) Ariz HPA, (7) Dareh Anjir WR. This figure was produced using ArcGIS (version 10.3.1 [www.esri.com/software/Arcgis]).

The cheetah (*Acinonyx jubatus*) is one of the most threatened carnivores at global level. Historically, it was widespread across Africa and southwestern Asia but it was extirpated from 91% of the former range due to habitat transformation, degradation and fragmentation, and depletion of its prey items^5^. The range decline was most severe in Asia: it was extirpated from almost all of its historical range and its distribution has been limited to the central plateau of Iran since the 1970s^6^. Within Iran, the Asiatic Cheetah (*Acinonyx jubatus venaticus*) has lost more than 70% of its suitable habitat over the past decades due to habitat transformation and loss of most of their main natural prey^7^. The population declined from over 200 individuals distributed across 44 areas in the mid-1970s to 50–100 individuals distributed in 14 areas in the 2000s^8,9^. Currently, the population is estimated at about 50 individuals distributed in three subpopulations located in the central plateau of Iran, encircling the Dasht-e Kavir desert (Fig. 1). The population is continuously declining and is divided into three subpopulations with an average distance of about 500 km between them: (1) Northern Subpopulation, (2) Southern Subpopulation and (3) Kavir Subpopulation; although since 2013, there have been no confirmed observations of Kavir Subpopulation^10^. Estimated boundaries of each subpopulation are shown by dashed lines in Fig. 1. The protected areas labelled in Fig. 1 constitute the last stronghold of the Asiatic cheetah and need an urgent boost in conservation efforts to prevent further population decline. The remaining Northern and Southern Subpopulations inhabit distinct habitat types. In general, the Northern Subpopulation occupies flat arid lowlands and the Southern Subpopulation ranges over mountainous deserts (Table 1). Consequently, prey abundance and availability are rather distinct in each of the regions, and potential regional differences in diet composition may be expected. As such, it is crucial to understand whether there are regional differences in diet composition and if diet in each region is reflecting prey abundance. Such information will allow defining conservation actions tailored to each region.

**Table 1:** Terrain roughness index (TRI), Altitude (m), Annual Precipitation (mm), Area (km^2^) of each study area, including mean, [minimum–maximum], and (standard deviation).

Area	TRI*	Altitude (m)**	Annual Precipitation (mm)**	Area (km^2^^2^)
Touran (N)	16.0 [0.0–294.3] (22.4)	932 [698–2,240] (236.3)	193.2 [155–247] (16.6)	14,415
Miandasht (NE)	12.0 [0.1–80.6] (11.4)	969 [888–1,216] (61.3)	274.2 [260–289] (6)	844
Naybandan (SE)	21.9 [0.0–312.3] (22.5)	1,040 [579–2,791] (201.3)	110.9 [89–130] (8.3)	15,170
Yazd (SW)	43.9 [3.4–409.4] (39.2)	1,399 [825–2,677] (320.6)	98.6 [87–118] (6.5)	4,475

*Extracted from Wilson et al.^92^; **Extracted from Fick and Hijmans^93^.

Subpopulations are indicated as Northern (N), North-eastern (NE), South-eastern (SE) and South-western (SW).

Most information on cheetah’s diet composition comes from Africa, where cheetahs have been reported to prey on a large variety of prey items, from large ungulates to small rodents^11^, preferring small to medium sized ungulates with body masses in the range of 15 to 65 kg, with the mode of 36 kg^12,13^. When these prey items are not available, such as outside birth peaks of ungulates or areas with lower ungulate population, cheetahs rely on smaller prey items such as hares (Leporidae)^14,15^. Cheetahs also predate on livestock, including sheep, goat and camel^16,17^, although they primarily consume wild prey^15,18^. Prey choice depends on prey abundance, presence of competitors, and cheetah’s sex^19^. Male coalitions are able to kill larger preys^20^, solitary females usually hunt smaller preys^13,21^, but when they have dependent cubs, they may take larger prey items^22^. In Iran, a few regional studies assessing cheetah’s diet reported jebeer (*Gazella bennettii*), goitered gazelle (*Gazella subgutturosa*), mouflon (*Ovis vignei*), ibex (*Capra aegagrus*), wild boar (*Sus scrofa*) and livestock (sheep, goat and dromedary) as main prey items^23–26^. Given that one of the most important cheetah refuges in central Iran, the Touran Biosphere Reserve, is inhabited by more than 70,000 livestock (sheep and goat) during the grazing season, knowing whether cheetahs regularly predate on livestock is vital to mitigate possible human-wildlife conflicts.

Diet analyses based on the morphological identification from scats are prone to errors regarding the identification of both, scat depositors and prey items^27,28^. Given that cheetahs start consuming their prey with muscle and viscera before chewing on skin and bones^29^, this can lead to biased estimations of consumed prey, e.g. to overestimate small sized prey and underestimate large sized prey^30,31^. Therefore, assessing cheetah diet using scat analyses, needs more accurate methods. Recent advances in DNA metabarcoding allow gathering accurate data on species’ diet, from bats to large mammals^32–34^. The method has been widely used in ecological studies, addressing the diet of carnivores^35,36^. Use of blocking primers can increase diet data acquisition by reducing amplifications from the predator^37^. The efficiency of using scat DNA metabarcoding for the analyses of cheetah diet using feeding trials was recently evaluated, reporting that it is possible to obtain diet information from 60-day old scats^38^. DNA metabarcoding from scat has been tested using wild cheetah scats in Africa and was reported to be a valuable complement to traditional dietary analysis methods, especially for items that might be missed by direct observation of killing events^17^. Patterns of prey selection may be different among individuals of the same species^39,40^ therefore individual identification and assessment of individual diet can provide important insights on population dynamics and conservation ecology^41^.

The main aim of this study is to provide accurate data on the foraging ecology of Asiatic cheetah based on scat DNA metabarcoding to support the definition of conservation measures, e.g. to identify the most important prey items for cheetah and boost their conservation. This is the first study assessing Asiatic cheetah diet using this approach and thus provides baseline methods and data that can be used as a guideline for future ecological surveys. This study aims to answer the following questions: What is the overall diet composition of Asiatic cheetahs? Is livestock consumed and do all individuals prey upon sheep and goats? Are there differences in diet between regions? Is diet composition correlated with habitat type and prey population size? Are there differences in individual dietary? The results of this study are intended to contribute to the conservation of the remaining critically endangered population of Asiatic cheetah in Iran.

## Results

From 376 collected scat samples, 138 (37%) were confirmed to be from cheetah (Table 2). Other predators found were common fox (*Vulpes vulpes*; 22%), unidentified Felidae (15%), wolf (*Canis lupus*; 16%), caracal (*Caracal caracal*; 6%), porcupine (*Hystrix indica*; 1%), and striped hyaena (*Hyaena hyaena*; 1%). Yazd had the highest percentage of confirmed cheetah scats (66%), while Miandasht with 7% had the lowest, and in Naybandan and Touran, the percentage was 33% and 39%, respectively. Cheetah scats were most frequently confused with fox scats in all areas, except in Miandasht where they were most often confused with other felids and Canis lupus (30% and 36%, respectively). Identification to species level in many specimens of Felidae family was not possible, neither to discern whether *Canis lupus* samples were from wolf or dog (*Canis lupus familiaris*), as the metabarcodes chosen in this work do not disambiguate these taxa.

**Table 2:** Identity of scat depositors, number of occurrences (N) and percentage (%) in total and in each study area.

Scat depositor	Total		Touran (N)		Miandasht (NE)		Naybandan (SE)		Yazd (SW)	
	N	%	N	%	N	%	N	%	N	%
*Acinonyx jubatus*	138	37	70	39	6	7	13	33	49	66
*Vulpes vulpes*	82	22	43	24	11	14	18	45	10	14
Felidae	58	15	30	17	24	30	2	5	2	3
*Canis lupus*	59	16	19	10	29	36	3	8	8	11
*Caracal caracal*	22	6	12	7	5	6			5	7
*Hystrix indica*	5	1	1	1			4	10		
*Hyaena hyaena*	4	1	4	2						
Unidentified	8	2	2	1	6	7				

Subpopulations are indicated as Northern (N), North-eastern (NE), South-eastern (SE) and South-western (SW).

We were able to genotype 100 of the original 138 cheetah scat samples and to identify a total of 14 individual cheetahs (3 females and 11 males): 2 females and 5 males in Touran, 1 male in Miandasht, 2 males in Naybandan (of which, one was also identified in Touran) and 4 males and 1 female in Yazd. We assessed diet composition from 119 out of 138 scats. We were unable to genotype 38 scats and assess the diet of 18 scats, due to very low quality of samples. A total of 13 distinct food items (species or genus) were observed in these scats. The main food items were mouflon (67%), ibex (26%), Cape hare (*Lepus capensis*; 13%), goitered gazelle (3%), wild boar (3%) and dromedary camel (*Camelus dromedarius*) (3%) (Table 3). Other food items with low occurrence were Midday jird (*Meriones meridianus*; N = 2), house mouse (*Mus musculus*), common fox and food items from genus *Jaculus* and orders Galliformes, Squamata and Chiroptera (N = 1 for each). On average, each scat contained 1.2 food items. Touran exhibited the highest richness of food items (N = 10). Regional accumulation curve of species observed in the diet is presented in Supplementary Fig. 1. Details of food items consumed by each genotyped individual are provided in Supplementary Tables 1 and 2. Given the low number of female cheetahs detected (N = 4), it was not possible to statistically compare the diet variation between different sexes. Comparing the diet of males and females shows females tend to predate on smaller preys such as cape hare, although they are capable of hunting larger preys, especially in more rugged areas such as Yazd. Males tend to predate on larger preys, such as mouflon and ibex, and dromedary camel was taken only by males. The male coalition in Yazd had the higher percentage of preying ibex in comparison to the males from other areas. Inferences of individual dietary differences are presented in Supplementary Table 2.

**Table 3:** Number of scats containing each food item (N), relative frequency of occurrence (RFO), prey population size (PS) and prey density (PD) (prey population/km^2^) for each prey item, in total and in each study area.

Food item	Total			Touran (Northern Subpopulation)				Miandasht (North-eastern Subpopulation)				Naybandan (South-eastern Subpopulation)				Yazd (South-western Subpopulation)			
	N	RFO	PS (N)	N	RFO	PS (N)	PD	N	RFO	PS (N)	PD	N	RFO	PS (N)	PD	N	RFO	PS (N)	PD
*Ovis vignei*	80	54	3,525	42	58	1,200	0.45	–	–	25	0.32	10	71	1,400	1.28	28	52	900	0.77
*Capra aegagrus*	31	21	3,400	8	11	700	1.62	–	–	–	–	1	7	1,500	1.82	22	41	1,200	0.76
*Gazella subgutturosa*	4	3	1,950	0	–	500	0.39	4	57	700	1.5	–	–	–	–	0	–	–	–
*Gazella bennettii*	0	–	900	0	–	150	0.12	0	–	–	–	0	–	200	0.78	0	–	60	0.09
Small livestock (sheep and goat)	0	–	–	0	–	76,000	7.6	0	–	15,000	17.75	–	–	–	–	–	–	–	–
Large livestock (dromedary camel)	4	3	–	1	1	1,500	0.15	–	–	400	0.47	2	14	1,500	0.10	1	2	–	–
*Lepus capensis*	16	11	–	13	18	–	–	1	14	–	–	1	7	–	–	1	2	–	–
*Sus scrofa*	4	3	–	2	3	–	–	1	16	–	–	–	–	–	–	1	2	–	–
N food items	14			10				4				4				6			
N scats	120			59				6				12				43			
N Occurrences	149			72				7				14				54			
Average N food items/scat	1.2			1.2				1.2				1.2				1.3			

*”N food items” is total number of food items found in each study area; “N scats” is the total number of scats from each area with identified prey items; “N occurrences” is the total number of occurrences of each prey items in all scats from each study area; “Average N food items/scat” is the number of food items in each area divided per number of scats in that area. Prey population size was extracted from DoE unpublished reports and enquiries from managers of protected areas in 2017. Prey density was calculated by dividing prey population by the distribution range of target prey species based on Yusefi et al.^67^.

In total and in each of the analysed areas, except Miandasht, mouflon was the most common food item found in scats, followed by Cape hare in Touran, dromedary camel in Naybandan, and ibex in Yazd (Table 3). Cape hare was the only food item that was observed in scats from all areas. Wild boar was detected a few times (total N = 4) in all areas except Naybandan. Despite being present in Touran and Miandasht, goitered gazelles were observed in the diet only in Miandasht, where they had the highest percentage of consumption (RFO = 57) jebeer was never observed in the analysed scats.

There were differences between prey abundance and prey consumed when analysing individual areas (Table 3). Mouflon was the most consumed food item in Touran, Naybandan and Yazd (RFO = 58, 71 and 52, respectively) and it was the food item with highest population size in Touran, while in Naybandan and Yazd, ibex has the highest population size. Ibex was observed only one time in scats from Naybandan, despite having larger population size than mouflon. In Yazd, where population of ibex is higher than mouflon, more scats contained ibex, but still the percentage of scats containing mouflon was higher (RFO = 52 for mouflon and 41 for ibex). Even though goitered gazelle have relatively large numbers in some parts of Touran, they were only observed in the diet of individuals from Miandasht. Visual comparison of relative percent consumption of different species to relative abundance of prey in each area is presented in Supplementary Fig. 2. Despite the large abundance of small-sized livestock, especially in the Northern Subpopulation, it was not found in any of the scats. Dromedary camel was rarely detected (total N = 4) in scats from Naybandan and Touran, where it is being kept as livestock.

## Discussion

In this study, we collected scats from all cheetah populations within the extant distribution range of the species in Asia, identified some different cheetah individuals, and analysed their diet using non-invasive and metabarcoding methods. Our study shows that cheetahs tended to hunt mouflon in study sites with predominantly more hilly terrains and gazelles in study sites that were predominantly flatter; small livestock was not detected in their diet. These findings have implications for cheetah conservation, as discussed in the following sections.

### Diet composition

We identified mouflon, ibex, cape hare, goitered gazelle, wild boar, and dromedary camel (in decreasing order of importance) as cheetah’s main food items. In comparison to other studies in Iran^23–26^, small livestock and jebeer were not detected in our results. This discrepancy could partially be due to misidentification of scat depositors in previous studies, as they were only based on the morphological identification of scats and could be partially due to low number of samples in this study, especially in Miandasht and Naybandan. The visual identification of faeces in diet composition studies has limitations, namely regarding the misidentification of scat depositors^27,42^ which can bias the results and misinform conservation managers^28,43^. For instance, Asiatic cheetah faeces used in the previous studies may have been confused with scats from leopard (*Panthera pardus*), wolf or hyaena, which are also present in the region (Table 2).

Overall, mouflon was the most consumed food item in Iran. Likewise, other studies also identified mouflon as the most consumed food item in the Southern Subpopulations^23,25,26^. In Africa, cheetahs mainly consume steppe-dwelling gazelles, such as impala (*Aepyceros melampus*) and Thomson’s gazelle (*Eudorcas thomsonii*)^44,45^. However, our results show that Asiatic cheetahs mainly take mouflon, which inhabits hilly and mountainous areas, rather than gazelles that inhabit plains. Given that Asiatic cheetahs are 30% smaller than South African ones^46^ and considering that preys such as Thomson’s gazelles are known as their preferred prey in Africa, it would be expected for jebeer and goitered gazelle to be the primary prey for the Asiatic cheetah. Indeed, previous studies conducted in 1970s to 2000s in Iran reported gazelles as cheetah’s primary prey item^47–49^, and recent studies found jebeer as the most preferred food item in Yazd-Naybandan^25,26^. Higher proportion of mouflon in the diet instead of the expected gazelles can be due to the severe declines in population range and size that cheetah experienced over the last decade, where most populations have been extirpated from their preferred plains, the preferred habitat of gazelles^7^.

We found that the diet composition apparently varies between geographic areas and these differences may be related with prey abundance and habitat characteristics. In some areas, cheetahs consume the most abundant prey, such as in Miandasht and Touran where they take goitered gazelle and mouflon, respectively. In other areas, the results shows that cheetah apparently consumes the second most abundant prey, such as in Naybandan and Yazd where they prey on mouflon rather than ibex (Table 3). Habitat characteristics are likely to interplay with prey availability: in comparison to ibex, mouflon inhabits less rugged areas, therefore in Naybandan, where rolling hills are more common than mountainous areas and TRI is lower than in Yazd, predation on ibex was only observed once. On the other hand, in Yazd where mountainous areas are the most common habitat type and TRI is higher than all the areas (Table 1), the proportion of ibex consumption was higher than in Naybandan (RFO = 41 in Yazd and 7 in Naybandan), similar to previous studies^23,25,26^. Although cheetahs are able to hunt successfully in various habitats^12^, their morphological adaption suggests that their primary habitat was open plains and that they evolved to hunt steppe-dwelling prey by chasing them in high speed, especially in open habitats^13,50,51^. In addition, old literature also suggested gazelles as their primary prey^47–49^. In Iran, when gazelles are unavailable, cheetahs need to hunt mouflon and ibex, which usually inhabit rugged mountains that are less suitable for cheetahs, where they might be even killed as competitors by leopards^7,52^. The absence of goitered gazelle in the scats analysed from Touran suggests that flat plains have become unsuitable for both cheetahs and gazelles, most likely due to poaching of gazelles and competition with livestock. Recent observations in Touran confirms that the goitered gazelles mainly occur in the vicinity of farmlands (HA personal communication), which are unsuitable for cheetahs due to disturbance associated with human settlements, transportation and livestock raising^7^. Although we sampled all known cheetah habitats in Yazd and Naybandan, jebeer was not detected in any of the analysed scats, possibly due to the severe decline in their population in recent years (Soofi et al., under review) and remarkable reduction in cheetah population size.

Small livestock was not detected in the diet analysed in our study, which contrasts with previous studies^24,26^, where domestic sheep and goat were detected as food items. This could be partially due to small sample size, especially in Miandasht and Naybandan. Despite large number of small livestock present in Touran and Miandasht and large herds of dromedary camel in Naybandan and Touran and smaller herds in Miandasht, sheep and goat were not detected in the analysed scats, and dromedary camel was detected only four times. Likewise, predation of Asiatic cheetahs on livestock was unreported in the old literature^48,49^. Therefore, the detection of small livestock as a food item in some recent studies^24–26^ may be the result of misidentification of scat depositors. In Africa, cheetahs prefer wild prey, but also consume small livestock, such as sheep and goat^15^. A possible reason for Asiatic cheetahs overlooking small livestock could be that livestock husbandry methods in Iran are an old practice where livestock herds are traditionally well protected by large guarding dogs that deter carnivores from approaching the herds^53^ and the smaller size of Asiatic cheetahs in comparison to southern African ones^46^. Still, the possibility of cheetahs occasionally consuming livestock is not fully rejected as there were evidences of predation on sheep in Chah-Shirin protected area (HA personal communication), suggesting that cheetahs may take small livestock in suboptimal habitats, with less wild prey^54^. As a matter of fact, we had only six samples from Miandasht which has the second highest livestock abundance, additional sampling may reveal consumption of livestock within protected areas. In addition, cheetahs may also consume dromedary, especially juveniles, as these are usually grazing freely with no shepherds or guarding dogs (B. Najafi personal communication).

Taken together, our results suggest that cheetahs in Iran tend to consume the most abundant wild prey, as long as the habitat characteristics make those prey items available. Moreover, our results can suggest that the recent decline in cheetah population^10^ and the shift in their distribution from flat areas to more unsuitable habitats^7^, such as hilly and mountainous habitats, may have induced also diet changes.

### Implications for cheetah conservation

Our results have important implications for conservation of Asiatic cheetahs. To prevent Asiatic cheetahs from extinction, making the plains suitable for them again and to ensure their long-term survival, we propose as conservation action the removal of livestock from cheetah habitats in Touran and Miandasht. Livestock husbandry and agriculture is historically the main livelihood of local people in central plateau of Iran, as consequence of several crop and animal domestication events in Fertile Crescent^55^. Therefore, although these areas are under legal protection by DoE, local people still have traditional rights to graze their livestock. Buying out grazing rights in Touran and Miandasht has long been proposed as a solution to remove livestock pressure from cheetah habitats^8^, was recently emphasized as an urgent conservation action and the priority pastures have been identified^10,56^. Similarly, removal of dromedary camel should also be prioritized as their large numbers can have wide negative impact in quality of grazing lands. Removing livestock will improve the quality of grazing lands, decrease livestock-wild herbivores competition, decrease human-wildlife conflict and poaching, and eventually allow gazelles to reoccupy the plains^57,58^. Removal of livestock will ultimately enhance feeding opportunities for cheetahs, thus promoting their long-term survival^44,59^. In Naybandan and Yazd areas, protection of mountain ungulates should continue but at the same time, the conservation of plains should be boosted to recover the population of gazelles.

### Sampling and barcoding constraints and innovations

The observed high confusion rate of scats with other predators (63%) emphasizes the importance of the genetic identification of scat depositors for complementing expert identification of scats in the field and for obtaining accurate data on the prey composition and predator community of each area. Several studies reported use of scat dogs as a complementary method to increase the probability of detecting scats in the field^60^. Using these dogs allows covering larger study areas and also decreasing laboratory costs by collecting scats from target species, and increasing the possibility of finding small scats from juveniles and females^61^. The problem of small number of samples size, especially in Miandasht and Naybandan can be overcome by using these dogs.

In our field work, we found scats from 11 male cheetahs but only from 3 females. The collection of scats in the field is usually biased towards male individuals, as they tend to mark their territories using scats^29^. Even though our samples were biased to male individuals, broad sampling (e.g., collecting both small and large-size scats) and sampling through line transects along previously known marking sites (instead of limiting our searches to marking sites) allowed us to obtain samples from four different females. The rate of identifying scat depositors’ species, and the individual and prey identifications using metabarcoding was high (98%, 72% and 87% respectively), which is probably due to low humidity of habitats, resulted in quick drying of scats and hence well preservation of scat’s DNA. The dry conditions of arid habitat make them very suitable for studies using non-invasive DNA.

The prey abundance data used in this study was obtained from annual census reports of Department of Environment. Although these data provide a coarse basis for estimation of prey abundance, it was not always collected with a standardize protocol. Therefore, for future studies, systematic sampling of prey abundance in all cheetah occurrence areas, preferably at the same time of sampling scats, is recommended to estimate prey abundance, which will allow estimating prey preference according to prey abundance. Systematic sampling of scats during several seasons to account for seasonality differences and retrieving all possible prey items and to prevent scat disappearance due to climate factors is also recommended^38^.

This was the first study using cheetah blocking primer to reduce predator amplifications from the diet analysis and better identify prey items. Although cheetah amplifications were not totally eliminated, they were decreased enough to allow obtaining more amplifications from the prey barcoding marker. The laboratory and bioinformatics pipeline presented in this study is an efficient and accurate tool to assess diet of large carnivores, especially in dry habitats. Using of molecular methods, allowed us to assess different ecological aspects of cheetah’s feeding ecology (e.g., sex and individual ID) which otherwise was not achievable. Therefore, we suggest to potential funders and donors to consider supporting molecular studies as a complementary part of field studies. To improve this pipeline, using additional markers to identify specimens of Felidae family to species level and to differentiate wolf and dog is recommended^62,63^ in order to have a better understanding of predator community of each area. Moreover, we recommend the development of a correction factor using feeding trials with captive animals to account for factors, such as meal size, prey species, feeding day, in order to obtain accurate data from the samples collected from the field.

### Implications for carnivore conservation

Our study highlights the usefulness of using genomic techniques along with well-established methods of data collection (e.g., camera trapping) as a complement for answering ecological questions. These genomic methods allow complementing current field assessments and also to provide information on different ecological aspects that are important for wildlife conservation. They also help managers to make informed decisions so far difficult to base on traditional methods. For instance, scats collected from other predators give a better understanding of competitor community and how they may compete with each other over available prey; individual identification data can give insights on sexual and ontogenetic differences of tropic niches and even differences between individual hunting strategies. Management plans for conservation of large predators can be improved by understanding adequately the exact predator–prey interactions, and understanding problems that they may cause, such as predation on domestic animals^64^. Thus, these data can improve conservation plans for many endangered carnivores, such as leopard, snow leopard (*Panthera unica*), or tiger (*Panthera tigris*), by e.g. identifying problematic predators responsible for livestock killing^65^, assessing their diets, investigating levels of human-wildlife conflict, and providing suggestions to decrease these conflicts.

## Methods

### Study area

The study area covers the protected areas with confirmed cheetah observations in 2017, distributed in the central deserts of Iran. In this study, the Northern Subpopulation was further divided into Northern Subpopulation covering Touran National Park (NP), Protected Area (PA) and Wildlife Refuge (WR), (hereafter Touran); and North-eastern Subpopulation comprising Miandasht WR and Zamen-e Ahoo NP, (hereafter Miandasht). The Southern Subpopulation was further divided into South-eastern Subpopulation covering Naybandan WR; and South-western Subpopulation comprising Kamki Bahabad Hunting Prohibited Area (HPA), Bafgh PA, Ariz HPA and Dareh Anjir WR, (hereafter Yazd) (Fig. 1). These areas are mostly covered by desert and semi-desert habitats and exhibit regional differences in land-cover and prey population size and availability: (1) Touran, is mostly flat and rangelands are the most common ecosystem, it displays a variety of habitats including a saline river system, mountain systems with high peaks and vast sand areas, including moving dunes and salt flats contiguous to Dasht-e Kavir; (2) Miandasht, comprises flat plains interrupted by rolling hills and a seasonal river; (3) Naybandan, comprises mostly plains, with hilly and mountainous areas with high peaks, vast salt plains and moving sand dunes; and (4) Yazd, is the most mountainous area, comprises mostly mountainous habitat and rocky hills with some low and rolling hills^66^. Miandasht, with a maximum terrain roughness index (TRI) of 80.6 (SD = 11.4), is the only flat region. The other three regions contain more mountainous terrain and have higher TRI and altitude differences. Among them, the Yazd areas has the highest TRI difference (SD = 39.2) and highest mean altitude (1399 m). Consequently, prey populations are rather distinct in each of the regions. In terms of wild prey, the most abundant prey in Miandasht is goitered gazelle and in Touran is mouflon, while in Naybandan, the most abundant prey is ibex, followed by mouflon and jebeer. Finally, ibex and mouflon are abundant prey items in Yazd along with very few gazelles^67,68^. In terms of livestock presence, very large numbers of small livestock (sheep and goats) are found in the range of the Northern Subpopulation, while Southern Subpopulations are only hosting few herds^69^; about 1,500 dromedary camel graze freely in Naybandan, about the same number in Touran and about 400 in Miandasht but few are found in Yazd areas (Table 3).

### Sample collection

Scat samples available for this study had two origins: (1) between May to September 2017, LK, EH, HA and TGH performed line transects across all protected areas to collect samples (N = 355). Surveys targeted trails, riverbeds, and shrub communities in the vicinity of previously known cheetah marking sites or other potential marking sites, such as large trees and big rocks. Given that the identification of large carnivores' scats in the field is prone to mistakes, we aimed for broad sampling, excluding only those prominent white scats from striped hyaenas (*Hyaena hyaena*)^70^, to include all potential cheetah scats; and (2) between 2014 and 2017, Conservation of Asiatic cheetah project (CACP) project members collected samples (N = 21) from Touran BR. In total, 376 putatively cheetah samples were available from Touran (181), Miandasht (81), Naybandan (40) and Yazd (74). Given the very low humidity of these areas, all collected samples were completely dry, thus they were stored in individual Ziploc bags, and then stored at room temperature until being transferred to the laboratory, where they were stored at − 20 °C until extraction. The fieldwork in the protected areas, collection of the samples and transferring them to the laboratory, were carried out under the permission from CACP. The geographic location of all samples was recorded with a Global Positioning System device on site in the WGS84 datum. Additionally, 15 tissue and hair samples of wild and domestic sheep and goats were collected to supplement the sequence database retrieved from NCBI (see “Laboratory analysis” section and Supplementary Table 3).

### Laboratory analysis

**Figure 2:**
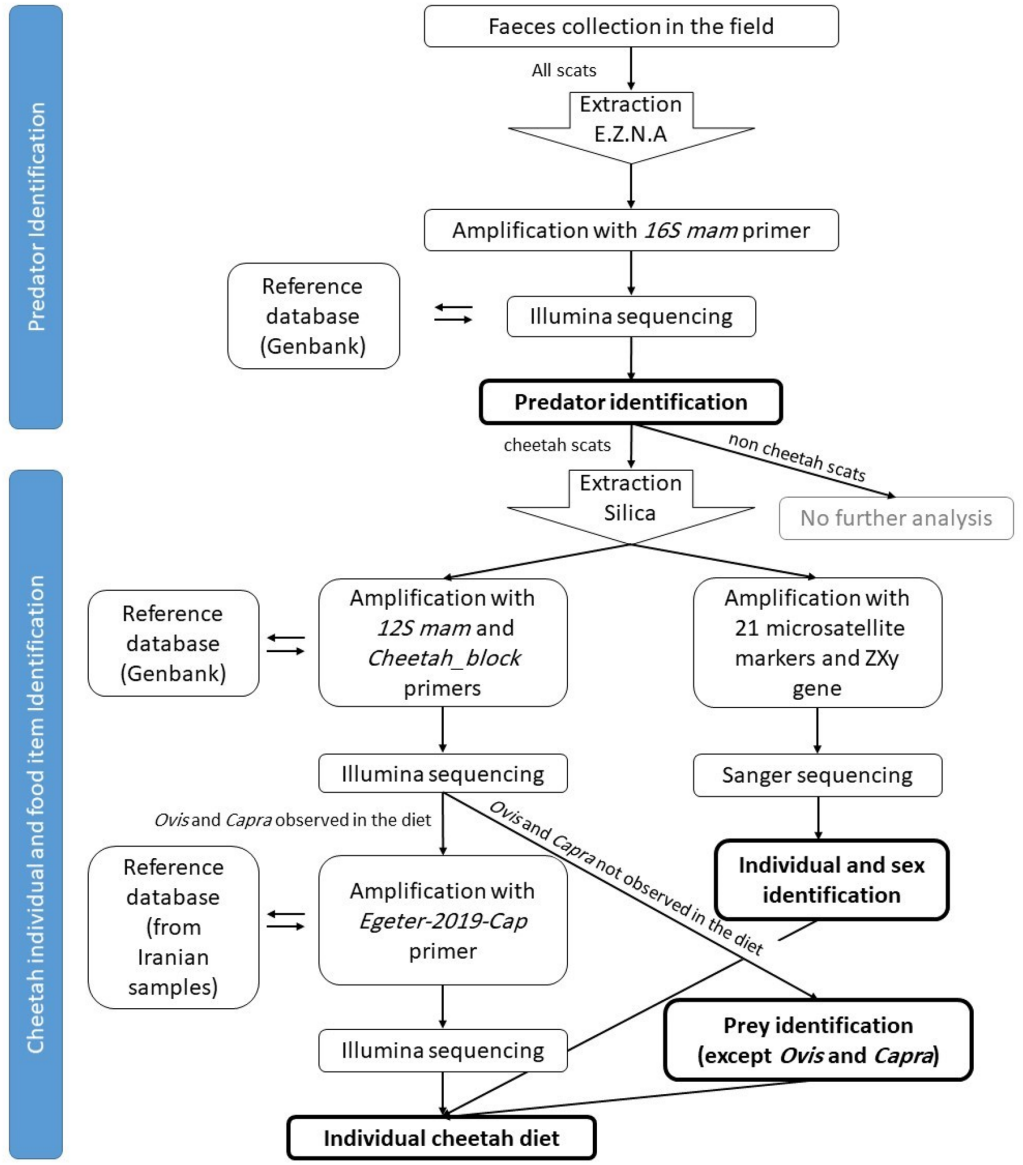
Flowchart diagram, showing the steps of laboratory analysis. Results of steps in boldface are reported in the result section, details on the results of other steps can be found in the Supplementary Material.

The first step for the molecular analysis was the identification of the depositor of each scat sample (Fig. 2). The E.Z.N.A Tissue DNA Kit (Omega Bio-tek, USA) was used to extract DNA from each scat sample following the manufacturer’s instructions, with the following modifications: manufacturer's TL buffer was replaced with Gordon’s lysis buffer (0.1 M Tris–HCl, 0.1 M EDTA, 0.01 M NaCl, 1% N–lauroyl sarcosine, pH 7.5–8^71^) and half of an inhibitEX® tablet (QIAGEN, GmbH, Hilden) was added after the first lysis step and before adding the OB protease to remove potential PCR inhibitors. Samples were extracted in batches of 24, each batch including a negative extraction control. Following extraction, DNA was amplified using the 16S_mam primers^72^ (Supplementary Table 4), modified to contain Illumina adaptors to allow the posterior addition of sample-specific barcodes. The PCRs were carried out in a final volume of 10 μl, comprising 5 μl of Qiagen Multiplex PCR Master Mix (Qiagen, Germany), 0.3 μl of each 10 pM primer and 2 μl of DNA extract. Cycling conditions were: initial denaturation at 95 °C for 15 min; 40 cycles of denaturation at 95 °C for 30 s, annealing at 52 °C for 30 s and extension at 72 °C for 30 s; a final extension at 72 °C for 10 min. Amplification success was checked by visually inspecting 2 μl of each PCR product on a 2% agarose gel stained with GelRed (Biotium, USA). Successfully amplified samples were then prepared for Illumina sequencing and sequences were blasted against the NCBI nt database to identify the predator species (see “Library preparation” and “Bioinformatics pipeline” sections).

The second step was the identification of food items in each scat sample (Fig. 2). From the samples that were confirmed to be of cheetah origin, DNA was extracted again following the protocol of Frantz et al. (2003)^73^ after applying the GuSCN /silica method^74^. As this method uses bigger portions of the scat, it allowed for DNA with better quality and quantity and minimising subsampling bias that can occur when smaller portions of scats are sampled^75,76^. Samples were extracted in batches of 32, with each batch including a negative extraction control. DNA was amplified using 12S primers^77^ (Supplementary Table 4; herein referred to as 12SV5.1), modified to contain Illumina adaptors. PCRs included a newly developed cheetah blocking primer for this primer set (Supplementary Table 4). PCR composition and conditions were the same as in the previous step but using an annealing temperature of 47 °C. Successfully amplified samples were then prepared for Illumina sequencing and sequences were blasted against the NCBI nt database to identify prey species (see the Library preparation and Bioinformatics pipeline sections). In both steps 1 and 2, to minimise the potential for contamination, extractions and manipulation of scat samples were carried out in a positive-pressure laboratory (CIBIO-InBio, Vairão Campus, Portugal), physically separated from other laboratory rooms that handle already extracted DNA material and PCR products, following strict protocols including disposable laboratory wear, UV sterilization of all equipment before entering the room, and cleaning laboratory surfaces with bleach between extraction batches. Instruments were sterilised by ethanol flaming twice between each sample. Additionally, each PCR plate included at least one PCR negative control to monitor for potential contaminations.

The third step consisted of distinguishing domestic from wild *Ovis* and *Capra* food items (Fig. 2). The metabarcode targeted by the 12SV5.1 primer pair does not provide resolution to achieve this. Instead, the mtDNA control region (D-Loop) was chosen for this aspect of the study, as this has been used previously to distinguish subspecies and lineages of goats^78^. A database was created comprising over 600 sequences of the control region from Iranian sheep and goats, mainly retrieved from NCBI, supplemented with 15 sequences obtained from tissue and hair-derived DNA extracts collected from wild and domestic sheep and goats in Iran. Each database entry was checked according to the data from original paper and designated as “Ovis wild”, “Ovis domestic”, “Capra wild” or “Capra domestic” (see Supplementary Table 3). Primers were designed to amplify a 100–122 bp fragment (excluding primer binding sites) within the region amplified by the CAP-FI and CAP-RI primer pair used by Luikart et al. (2001)^78^. Primers included degenerate bases to facilitate amplification of multiple species of *Ovis* and *Capra*.

PCR composition and conditions were the same as in the previous steps, but with an annealing temperature of 45 °C and a PCR cycle number of 38. Successfully amplified samples were then prepared for Illumina sequencing and sequences were blasted against the custom database (see “Library preparation” and “Bioinformatics pipeline” section).

In the fourth step, we assessed the individual ID of cheetah samples, using a set of 21 microsatellites developed for *Felis catus*^79^ and tested on cheetahs^80–83^. DNA samples were amplified using a multiple-tubes approach so as to reduce the genotyping errors^84^, using four replicates. PCR products were separated by size on an ABI 3130xl DNA analyzer (Applied Biosystems, Foster City, California). Microsatellite alleles were scored using the GeneScan500 LIZ size standard and GeneMapper v4.0 (Applied Biosystems, Foster City, California), and checked manually. Multiplex 2 was used first on all samples to assess the quality and samples with more than 70% of amplification were chosen to test with remaining multiplexes. For sex identification, we used ZFx gene in Multiplex 2. For details on multiplex combination and Thermocycling parameters allele range see Supplementary Table 4.

### Library preparation

Amplicons from the initial PCRs from the first, second and third steps (see above) were prepared for sequencing using the Illumina platform following Egeter et al. (2018)^85^, with the following minor modifications: a ratio of 0.9 X AMPure XP beads (Beckman Coulter, USA) was used for the purification step; quantification of each purified PCR product was conducted using the EPOCH plate reader (BioTek, USA). Final pools were sequenced on either the Illumina Miseq (CIBIO, Portugal) or Hiseq (GeneWiz) platforms.

### Bioinformatic pipeline

Reads were demultiplexed according to the sample-specific indexes using BASESPACE (basespace.illumina.com). Sequence data were processed using the MBC pipelines package (Galhardo et al. in prep.; see Supplementary Methods for exact commands). Within the package, paired-end reads were aligned using flash2^86^ with the settings: –max-overlap = 100 -D -m 10 -t 1. Sequences outside the expected amplicon lengths were removed. Remaining sequences were demultiplexed using vsearch^87^ with fastq_maxee = 1 and singletons were removed. The zero-radius Operational Taxonomic Units (OTUs) were mapped against relevant databases, using the blastn algorithm, and 100 results per query were kept. To obtain full-length alignments only, rather than the partial alignments blastn can often return, the gap and extension penalties were relaxed and only alignments with a query cover of 98% were kept (see Supplementary Methods for exact commands). Blast results to the taxa that were not present in Iran^67^ were removed.

OTUs were placed in taxonomic bins using the metabin program in metabinkit (v0.1.0; https://doi.org/10.5281/zenodo.3855032). To choose taxonomic thresholds we: 1) measured the within-taxon and among-taxon percentage identity variation for all taxa in the blastn results, and; 2) run metabin numerous times with varying thresholds to ensure that the correct assignments were being retrieved for each database entry (see Supplementary Methods for more details). The final percentage identity taxonomic thresholds were species = 99%, genus = 99%, family = 99% and above-family = 99%. Within metabin mammal species that do not occur in the study area^67^ were disabled. To remove potential crosstalk between samples, detections that were < 0.1% of the total read count for the respective taxon were removed, as were detections that were < 0.1% of the total read count for the respective sample. To further remove any potentially spurious results, all detection < 40 reads were discarded. Detailed taxa-tables, produced from 16S_mam, 12SV5.1 and Egeter-2019-Cap primer, were presented in Supplementary Table 5, 6 and 7 respectively.

Consensus genotypes over four replicates were assembled following three rules: heterozygous genotypes were accepted if the same genotype was observed in two independent PCRs; homozygous genotypes were accepted if the genotype was observed in or three independent PCRs^88^; only one allele was accepted (and missing data for the second allele) at a locus when observed in each of two available replicates. We discarded replicate samples from analysis as determined by identical genotypes using Gimlet v1.3.2^89^. The same software was used to measure mean allelic dropout and false allele rates across loci, samples, and PCRs. Finally, we estimated the probability of identical genotypes being shared by chance (probability of identity, PID, and Probability of Identity among siblings PIDsibs) to assess if our markers’ resolution was sufficient to reliably differentiate among closely related individuals, including siblings.

### Statistical analysis

Given that the abundance data were collected using variable protocols (see “Sampling and barcoding constraints and innovations”), we were unable to estimate prey preference according to prey abundance, and instead we used more simple and descriptive analyses. To quantify Relative Frequency of Occurrence (RFO) of food items in diet composition, each prey species detected in one scat sample was assumed to represent a single predatory event^15^. Given that metabarcoding methods did not rely on visual identification of prey remaining, we did not used any correction factor^90^. The RFO is the relation of identified prey items to the number of all prey items found in all scats following Breuer 2005 and references therein^91^. We excluded food items that were observed in very low number of scats (< 3) or in very low reads in comparison to other food items. In the former case we consider that they were not repeated enough times to have significant impact on results, and in the latter case we consider that they could be the result of field contaminations (e.g., from prey's scat). RFO was calculated for each of the four regions and across the entire study area. Prey population size was extracted from DoE (Department of Environment of Iran) regional office census report of each province (Unpublished data, DoE). Given that cheetahs display large home-range sizes (1136 km^2^)^52^, all of each region was considered as hunting area of cheetah. Since we observed evidence of cheetah movements between protected areas in Yazd (i.e. identification of the same individual in several areas), all of them were considered as a single region.

## Supplementary Information

Supplementary Information.

## Data Availability

The raw sequencing data has been deposited at the European Nucleotide Archive (ENA) under the accession number PRJEB39665 (https://www.ebi.ac.uk/ena/browser/view/PRJEB39665).
